# Neutron reflectometry with the Multi-Blade ^10^B-based detector

**DOI:** 10.1098/rspa.2018.0266

**Published:** 2018-08-22

**Authors:** G. Mauri, F. Messi, M. Anastasopoulos, T. Arnold, A. Glavic, C. Höglund, T. Ilves, I. Lopez Higuera, P. Pazmandi, D. Raspino, L. Robinson, S. Schmidt, P. Svensson, D. Varga, R. Hall-Wilton, F. Piscitelli

**Affiliations:** 1 European Spallation Source ERIC, PO Box 176, 221 00 Lund, Sweden; 2 Department of Physics, University of Perugia, Piazza Università 1, 06123 Perugia, Italy; 3 Division of Nuclear Physics, Lund University, PO Box 118, 22100 Lund, Sweden; 4 Laboratory for Neutron Scattering and Imaging, Paul Scherrer Institute, 5232 Villigen PSI, Switzerland; 5 Department of Physics, Chemistry and Biology, Linköping University, 581 83 Linköping, Sweden; 6 Wigner Research Centre for Physics, Konkoly Thege Miklós út 29-33, 1121 Budapest, Hungary; 7 ISIS Neutron and Muon Source, Harwell Oxford, Didcot OX11 0QX, UK; 8 IHI Ionbond AG, Industriestrasse 211, 4600 Olten, Switzerland; 9 Mid-Sweden University, 851 70 Sundsvall, Sweden

**Keywords:** neutron detectors (cold and thermal neutrons), gaseous detectors, boron-10, neutron reflectometry, neutron scattering

## Abstract

The Multi-Blade is a boron-10-based gaseous detector developed for neutron reflectometry instruments at the European Spallation Source in Sweden. The main challenges for neutron reflectometry detectors are the instantaneous counting rate and spatial resolution. The Multi-Blade has been tested on the CRISP reflectometer at the ISIS Neutron and Muon Source in the UK. A campaign of scientific measurements has been performed to study the Multi-Blade response in real instrumental conditions. The results of these tests are discussed in this paper.

## 1. Introduction

The Multi-Blade [1–5] is a^10^B-based detector for neutron reflectometry instruments [6–8]. The detector requirements are set by the two reflectometers that are being designed for the European Spallation Source (ESS [9]) in Sweden: FREIA [10,11] (horizontal reflectometer) and ESTIA [12–14] (vertical reflectometer). In the past few years, several methods have been proposed to improve the performance of reflectometry instruments, and the ESS reflectometers are based on these new concepts.

Neutron reflectometry and off-specular scattering are powerful techniques to study depth profiles and in-plane correlations of thin film samples [15–17]. In a typical neutron reflection experiment the reflection of neutrons as a function of the wavevector transfer in the direction of the surface normal, *q*_*z*_, is measured:

1.1 $$q_{z}=\frac{4\pi }{\lambda }\sin(\theta ),$$

where λ is the neutron wavelength and *θ* is the angle between the beam and the sample surface (which is the same for the incident and the reflected beam, *α*_*i*_ = *α*_*f*_ = *θ*).

Neutron reflection follows the same fundamental equations as optical reflectivity but with different refractive indices. The optical properties of neutron propagation arise from the fact that, quantum mechanically, the neutron is described by a wave function. The potential (*V* ) in the Schrödinger equation, which is the averaged density of the scattering lengths of the material, plays the role of a refractive index,

1.2 $$V=\frac{2\pi \hbar^{2}}{m_{n}}N_{b}=\frac{2\pi \hbar^{2}}{m_{n}}\sum_{i}b_{i}n_{i},$$

where *m*_*n*_ is the neutron mass, ℏ is Planck's constant, and *N*_*b*_ is the *scattering length density* of the medium, where *n*_*i*_ is the number of nuclei per unit volume and *b*_*i*_ is the coherent scattering length of nucleus *i*, because we take the spin average (non-polarized beam or sample).

The neutron refractive index is given by the scattering length density of its constituent nuclei and the neutron wavelength. As with light, total reflection may occur when neutrons pass from a medium of higher refractive index to one of lower refractive index. The angle where no neutrons penetrate the surface, hence all of them are reflected, is called the *critical angle* (or equivalently the *critical edge*): the reflectivity of neutrons of a given wavelength (or given *q*) from a bulk interface is unity at smaller angles and falls sharply at larger angles. As with light, interference can occur between waves reflected at the top and at the bottom of a thin film, which gives rise to interference fringes in the reflectivity profile [15].

The typical neutron wavelengths (λ) in a reflectometry experiment are in the range 2–20 Å, which corresponds to a range between 0.05 and 3 nm^−1^ in the wavevector transfer (*q*_*z*_). In the real space this corresponds to length scales between 2 and 100 nm [18]. The limits are imposed both by the measurement range and by the instrumental resolution. In the case of off-specular scattering, it is possible to investigate objects in the plane with a correlation length of the order of several micrometres (50–0.5 mm). The upper limit is set by the resolution of the instruments and the size of the direct beam. The lower limit is determined by the available neutron flux [18].

In the last two decades reflectometers have been optimized and allow reflectivities to be measured below 10^−6^, which is sufficient for most experiments [19]. The next step is to increase the available flux; this leads to a significant speed-up of reflectivity measurements and the possibility of using smaller samples.

Several techniques have been recently proposed to improve the operating performance of reflectometry instruments. The methods are based on spin–space [20], time–space [21] or energy–space encoding [22–25]. The first technique is used for off-specular measurements [26] and encodes the incident angle by the rotation of the neutron spin in a magnetic field. The time–space encoding (TilTOF) enables an increase in the incoming flux on the sample, removing the chopper and modulating mechanically the angle of the sample to determine the time shape of the beam, and thus the wavelength. The idea of energy–space encoding is to analyse the neutron energies through a spatial spread of the reflected beam produced by an energy dispersive device, either a refractive crystal [24,25] or a magnetic field gradient [22]. It is also possible to correlate the neutron wavelength and the incident angle, before the sample, using a divergent beam focused on the sample. The REFocus [23] technique employs an elliptical graded multilayer monochromator to focus the neutrons on the sample. This concept has been modified and adapted to the time-of-flight (ToF) instrument AMOR at the Paul Scherrer Institute (PSI) [27], using an elliptic-shaped reflector: the *Selene* guide [13,28]. A scaled-down demonstrator is implemented on AMOR at PSI [14] to prove the concept and to test the performance of the guide. The full-scale Selene guide will be a primary feature for ESTIA, a reflectometer instrument at ESS, now under construction.

The general aim of all these optimizations is to increase the available neutron flux on the sample; thus time-resolved measurements for kinetic studies can be performed, smaller samples can be used and faster measurements scaling down from hours, the typical time for present-day reflectivity experiments, to minutes can be performed. This gives the possibility of probing a dynamic range of reflectivity measurements down to 10^−7^. Although these dynamic ranges can be achieved with current instruments, they are not routinely performed in most experiments, because the background affects the measurements significantly, and they require long data acquisitions.

These improvements represent a challenge not only for the instrument design, but also for the performance of the detector technologies to be employed. The current detector technology is reaching fundamental limits, e.g. a sub-millimetre spatial resolution (full-width half-maximum, FWHM) and high counting rate capabilities are required for the new instruments and these are not achievable with the state-of-the-art technology. Furthermore, the detector system should not limit the reachable dynamic range. It is also necessary to achieve an adequate signal-to-noise ratio, e.g. discriminating fast neutron and gamma radiation events [1,29].

The expected instantaneous local flux at the detector, i.e. after reflection from the sample, for the reflectometers at ESS is about 10^5^ n s^−1^ mm^−2^ [30–32]. In the case of ESTIA, considering a sample of 50 × 20 mm^2^ at an incident angle of 1.2°, the effective area would be 20 mm^2^ with 5 × 10^7^ n s^−1^ mm^−2^ at the sample position. This would reflect 10^9^ n s^−1^; however, the reflection will diverge at the detector to a size of ≈ 100 × 100 mm^2^. Thus, the count rate on the detector would be of the order of 10^5^ n s^−1^ mm^−2^.

Note that the current detector technology is already limiting the performance of the neutron reflectometers at existing sources (pulsed and reactors).

The Multi-Blade detector has been designed to fulfil these challenging requirements in terms of spatial resolution and counting rate capability. A demonstrator has been installed at the neutron reflectometer CRISP [33] at the ISIS Neutron and Muon Source in the UK [34]. The detector has been characterized and a series of scientific measurements with several samples have been performed. The technical characterization of the Multi-Blade is not treated in this paper; a detailed description can be found in [5]. The performance of the Multi-Blade detector concerning the scientific measurements is the subject of this paper. The aim of this test is not only to prove the capabilities of the detector in an actual instrument, but also to show the improvements that arise from operating the CRISP reflectometer in a configuration which reproduces the ESTIA operation mode. This is exclusively possible by exploiting the features of the Multi-Blade.

## 2. The multi-blade detector tested at crisp

**Figure 1.**
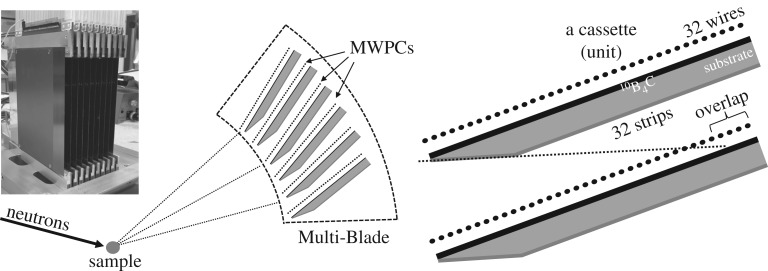
Schematic view of the cross-section of the Multi-Blade detector made up of identical units (cassettes) arranged adjacent to each other. Each cassette holds a^10^B_4_C layer; the read-out is performed through a plane of wires and a plane of strips.

The Multi-Blade is a stack of multi-wire proportional chambers (MWPCs) operated at atmospheric pressure with a continuous gas flow (Ar/CO_2_ 80/20 mixture by volume). A sketch of the Multi-Blade detector is shown in figure 1. The Multi-Blade is made up of identical units, the so-called ‘cassettes’. Each cassette holds a ‘blade’ (a flat substrate coated with^10^B_4_C [35–37]) and a two-dimensional read-out system, which consists of a plane of 32 wires and a plane of 32 strips. The wire pitch is 4 mm and the strip width is also 4 mm. Each^10^B_4_C converter (blade) is inclined at grazing angle (*β* = 5°) with respect to the incoming neutron beam. The cassettes are arranged over a circle around the sample and they have some overlap, i.e. each blade makes a shadow over the adjacent one in order to avoid dead areas. Two adjacent cassettes are not parallel; rather, they are placed at a relative angle of 0.14°.

The physical dimensions of a cassette are approximately 130 mm across both the wire and strip planes. Because of the inclined geometry, the projection of one cassette towards the neutron incoming direction (sample position) does not affect the strip plane, but results in a reduction of a factor sin(*β*) of the wire plane extension. Thus, the projected area of a cassette is approximately 11 × 130 mm^2^. The strip bin size remains at 4 mm, while the wire bin size is ≈ 0.35 mm (=sin(*β*) × 4 mm). Note that the bin size is not the spatial resolution of the detector and it is not only determined by the wire and strip pitch. The track length of the neutron capture fragments is comparable to the chosen pitch distance. The spatial resolution (FWHM) has been previously measured [1,2] and confirmed by the present test [5], scanning the active area of the detector, in both directions, with a collimated beam. The spatial resolution across the wire direction is between ≈ 0.5 mm and 0.6 mm, and across the strips is ≈ 3.5 mm. A detailed description of the detector can be found in [1,5].

The present detector consists of nine units (576 channels in total), corresponding to an active area of 100 × 130 mm^2^. Each channel (64 per cassette) is read out individually, connected to a field effect transistor-based charge pre-amplifier and shaping amplifier. Each 32-channel board is connected to a CAEN V1740D digitizer (12 bit, 62.5 MS s^−1^) [38]. There are six digitizers in total and each can read out 64 channels, i.e. one cassette. Thus, out of the nine cassettes, six could be used simultaneously in the tests, i.e. ≈ 70 × 30 mm^2^ active area. The six digitizers can be synchronized to the same clock source and a transistor–transistor logic signal can be sent to one of them and propagated to reset the time stamp which is associated with an event. This feature is needed to perform any type of ToF measurement. In the case of CRISP, the reset of the time stamp is given by the proton pulse of the ISIS source.

The raw data from the read-out electronic system are reduced to a triplet (*X*, *Y*, *ToF*), which identifies a single neutron event. The reconstruction algorithm used is described in detail in [5]. The triplets define a three-dimensional space containing the information, where in the detector the neutron was detected with associated ToF. The spatial coordinates, *X* and *Y* , of a triplet reflect the physical channels in the detector (32 wires and 32 strips) projected over the detector entrance window (i.e. the projection of the blades towards the sample position). The Multi-Blade detector is, indeed, a three-dimensional detector, but the depth coordinate (*Z*) is integrated over.

We assume that *X* is the horizontal coordinate and *Y* the vertical. Based on the geometry of the instrument, either a horizontal or a vertical reflectometer, the higher spatial resolution is needed in the vertical or in the horizontal plane, respectively. The Multi-Blade detector has the best spatial resolution across the wire plane. Thus, in a horizontal reflectometer, the *Y* coordinate represents the wires and the *X* coordinate the strips. In a vertical reflectometer this convention is inverted.

## 3. Experimental set-up on crisp

CRISP is a horizontal neutron reflectometer at ISIS, Target Station 1, that uses a broadband neutron ToF method to determine the wavelength (and hence *q*) at fixed angles (*θ*). A detailed description of the CRISP reflectometer can be found in [33]. The instrument views a hydrogen moderator, giving an effective wavelength range of 0.5–6.5 Å at the source frequency of 50 Hz. The wavelength band extends up to 13 Å if operated at 25 Hz. A frame overlap mirror suppresses the wavelengths above 13 Å. The distance from the moderator to the sample is 10.25 m and the sample to the Multi-Blade detector distance is approximately 2.3 m.

**Figure 2.**
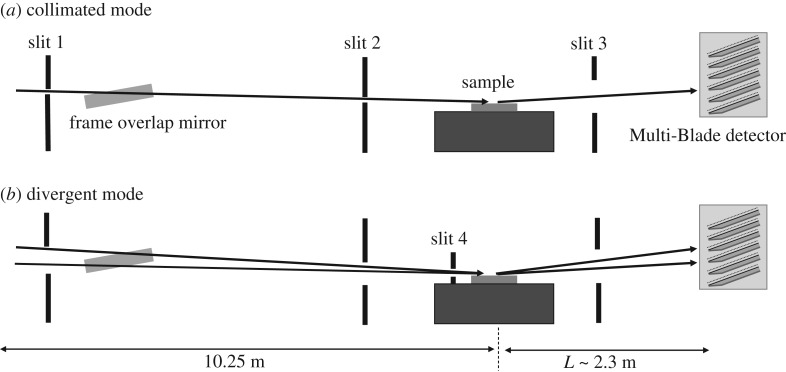
A sketch of the CRISP horizontal reflectometer and the Multi-Blade detector showing the orientation of the cassettes. The beam can be collimated at the sample position either with a low divergence (collimated mode (*a*)) or by allowing more divergence of the beam (divergent mode (*b*)).

**Figure 3.**
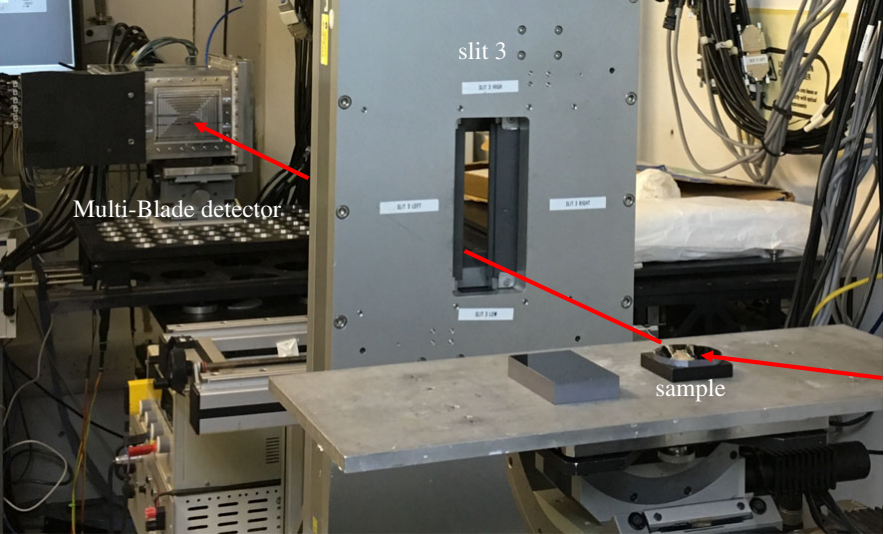
The Multi-Blade installed on the table of CRISP on a goniometer. A view of the incoming and the reflected beam reaching the active area of the Multi-Blade detector is shown. (Online version in colour.)

The beam can be well collimated using adjustable slits along the beam line; a sketch is shown in figure 2. According to the position and the opening of the slits, we performed the measurements in two working modes: collimated and divergent. In the collimated mode, the slits are set in order to achieve a good collimation of the beam at the sample. The divergent mode is obtained by opening as much as possible the slits before the sample. According to the concept of REFocus [23], proposed for ESTIA [13,28], one more slit with a narrow opening (≈1 mm) was added before the sample as shown in figure 2. Figure 3 shows the Multi-Blade installed on the CRISP reflectometer.

Three standard and well-known types of samples have been used in the tests: an iridium (Ir) sample, slightly bent, deposited on a silicon substrate (4 × 4 cm^2^); a bare silicon (Si) sample (≈8 cm diameter); and an Fe/Si super-mirror (≈4 cm diameter), which is used in neutron optics to deliver neutrons to the instruments. The Ir sample has been used to study the effect of the spatial resolution of the detector on the measured reflectivity curve and will be shown in §4a. The Si sample has been used to study the collimated and divergent modes. This will be discussed in detail in §4b. The Fe/Si super-mirror has been used to study the off-specular scattering with the Multi-Blade and will be discussed in §4c.

## 4. Results

**Figure 4.**
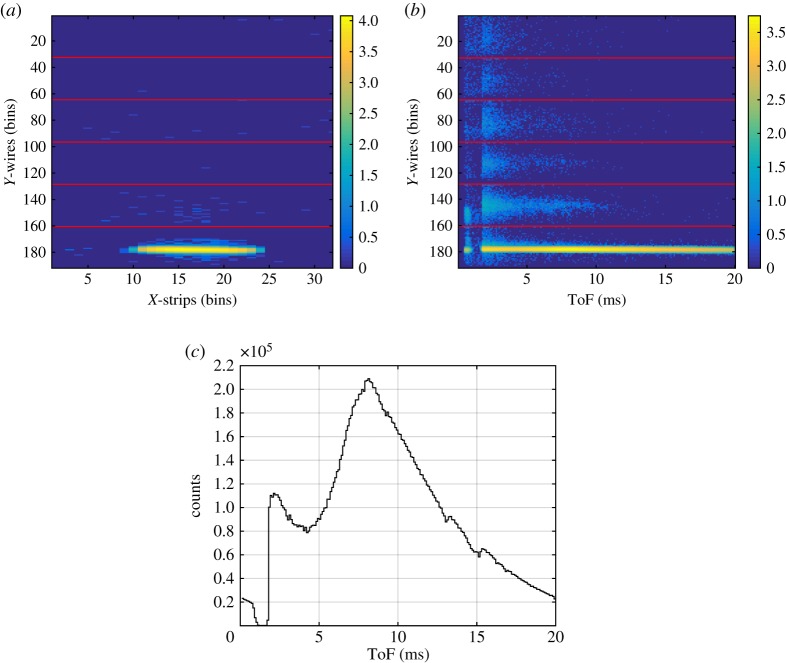
(*a*) Two-dimensional image of the direct beam impinging on the lower cassette of the detector. A gate in ToF, between 12.5 ms and 20 ms (4 Å –6.5 Å), is applied. The bin size on the *Y* -axis is 0.35 mm and on the *X*-axis is 4 mm. (*b*) ToF image of the detector integrated over the *X* coordinate. The bin size on the *Y* -axis is 0.35 mm and 100 ms on the *ToF* axis. The colour bar represents counts on a logarithmic scale. (*c*) Intensity of the direct beam in ToF, integrated over the *X*- and gated in the *Y* -coordinate.

The triplets (*X*, *Y*, *ToF*) that identify a neutron event can be represented by two-dimensional plots: the two-dimensional image of the detector reproduced by the (*X*, *Y* ) coordinates, and the ToF image of the detector which corresponds to the (*Y*, *ToF*) coordinates integrating over the other spatial coordinate (*X*). Moreover, the two-dimensional image (*X*, *Y* ) can be either integrated over the ToF coordinate or gated in any range of time. Also the ToF image can be integrated or gated over the spatial coordinates. A ToF of 6 ms corresponds approximately to 1.8 Å, 8 ms to 2.5 Å and 12.5 ms to 4 Å. An example of these plots is shown in figure 4 and corresponds to a measurement of the direct beam hitting the lower cassette. The two-dimensional image ((*X*, *Y* ) on a logarithmic scale) of the direct beam, gated in ToF between 12.5 ms and 20 ms (4 Å–6.5 Å), is shown in figure 4*a*. The horizontal red lines indicate where each cassette starts and ends. The ToF image ((*Y*, *ToF*) on a logarithmic scale) is shown in figure 4*b*. The ToF integrated over the *X* coordinate and gated in the *Y* coordinate around the direct beam area is shown in figure 4*c*. This is used to normalize the reflectivity measurements of the samples described in the following sections. The time binning of 100 ms was chosen to match the ISIS-Target Station 1 pulse length.

The gate in ToF is applied in order to reject the background arising from the spurious scattering from the substrate of the cassettes (the blades). This effect is due to the neutrons that cross the^10^B_4_C layer without being absorbed. They are scattered by the substrate and detected in the other cassettes. This background has been understood quantitatively and its full characterization is explained in detail in [5]. Although this effect can be minimized during the analysis by applying the gate in the ToF mentioned above, it can be avoided with technical measures that will be implemented in the next detector generation [5]. As mentioned above, the triplets define a three-dimensional space; the third coordinate (*Z*), which is integrated over, describes the physical position of each wire in depth. As this position is known, the flight path *D* can be corrected with the distance (*Z*_*i*_) of the *i*-th wire of each cassette according to the following formula:

4.1 $$D_{i}=D_{0}+Z_{i}=D_{0}+(Y_{i}-1)\times (p\times \cos(\beta )),$$

where *D*_0_ depends on the instrument geometry and, in our case, is the distance from the moderator to the first wire (front wire) of the Multi-Blade corresponding to *Y*_*i*_ = 1, *p* = 4 mm is the wire pitch and *β* = 5° is the inclination of each blade with respect to the sample position.

### (a) Specular reflectometry on Ir sample: improvement of the *q*-resolutionwith the detector spatial resolution

**Figure 5.**
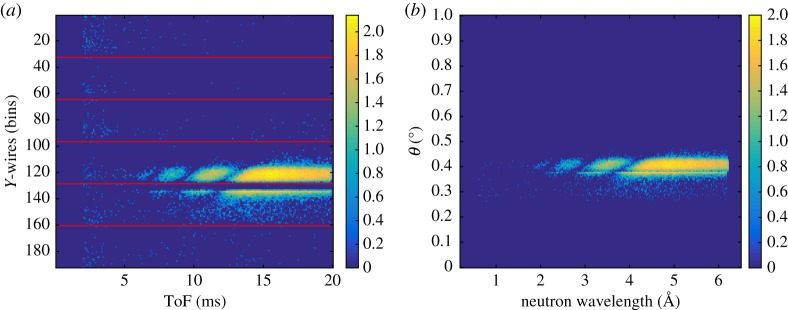
(*a*) ToF spectrum of the reflected beam from the Ir sample. The bin size on the *Y* -axis is 0.35 mm and 100 ms on the *ToF* axis. The horizontal lines depict the end of each cassette and the gap in between is the shadowing effect due to the geometric properties of the detector. (*b*) ToF spectrum reduced in the (*θ*, λ) space. The gap does not represent a dead area, and thus can be removed without losing information as shown for the reduced data in (*θ*, λ) space. The colour bar represents counts on a logarithmic scale.

An iridium (Ir) sample has been used to perform measurements of specular reflectivity. The aim of this measurement was to show how the data analysis can be improved, if the detector spatial resolution is taken into account, and how a finer spatial resolution affects the quality of the results. The reflected intensity from the Ir sample in the (*Y*, *ToF*) coordinates is shown in figure 5*a*.

The horizontal lines represent the boundaries of each cassette and the gap in between is a shadowing effect caused by the arrangement of the blades. Two subsequent cassettes are arranged in order to have an overlapping area; therefore, the gap is not a dead area of the detector. The last firing wire of one cassette, not necessarily the last physical wire, is, in the projected space (*X*, *Y* ), the neighbour of the first wire of the adjacent cassette. Thus the gap can be removed without losing any information. Moreover, because of the blade geometry the gas gain differs for different wires within a cassette, as shown in [1,5]. The gain drops in the first seven wires, but it can be compensated by adjusting individual thresholds on each channel. At the very first wire the loss in efficiency corresponds to a drop of 50% with respect to the nominal efficiency. This region of reduced sensitivity is where two cassettes overlap and it is about 0.5 mm wide, as shown in figure 1.

In figure 5*b*, the (*θ*, λ) phase space obtained from the (*Y*, *ToF*) space is shown. Note that, in this plot, the gaps have been removed and the sole reduced sensitivity area is still visible in the plot. The neutron wavelength (λ) is calculated from the ToF corrected according to the depth of the detector (equation (4.1)), thus the exact neutron wavelength can be calculated.

According to equation (1.1), the wavevector transfer *q*_*z*_ depends on *θ* (determined by the instrumental settings) and λ. The maximum intensity corresponds to the angle between the scattered beam and the sample, *α*_*f*_, being equal to the incident angle *α*_*f*_ = *α*_*i*_ = *θ*. According to the conventional analysis, for each wavelength, *q*_*z*_ is calculated with a fixed and unique *θ* following equation (1.1) and integrating the intensity over the full size of the beam. The width of the reflected intensity is defined in a range *α*_*f*_ = *θ* ± Δ*θ*. The latter originates from the divergence of the beam.

The spatial resolution of the detector can be used to include a correction over *θ*, as for a small projected sample size this position directly correlates with the reflection angle. This can be used to correct for the increased spread of the reflected beam caused by a slight bend of the sample surface, which would otherwise reduce the *q*-resolution. In contrast with the conventional analysis, each value of *q*_*z*_ is calculated according to its relative *θ*_*i*_ = *α*_*i*_ + *δθ*_*i*_ defined by the position on the detector. The correction is shown in equation (4.2):

4.2 $$\theta_{i}=\alpha_{i}+\delta \theta_{i}=\alpha_{i}+f\times \;\text{arctan}\;\left(\frac{(Y_{i}-Y_{0})\times p_{s}}{L}\right),$$

where *Y*_0_ is the position of the bin corresponding to *α*_*f*_ = *α*_*i*_, *Y*_*i*_ is any other position in the integration range, *L* is the distance between the sample and the detector (2.3 m), and *p*_*s*_ is the pixel size of the detector. Note that the pixel size of the Multi-Blade is *p*_*s*_ = *p* × sin(*β*) ≈ 0.34 mm, where *p* = 4 mm is the wire pitch, and is finer than the spatial resolution of the detector, which is ≈ 0.6 mm. The factor *f* = 1/2 has to be introduced, as the bending of the sample surface acts as a change in sample angle and leads to a change in reflection angle by 2*θ*. Different combinations of λ and *θ* correspond to the same *q*_*z*_ in a diagonal cut of the (*θ*, λ) space; this leads to an improvement in the resulting reflectivity profile.

Figure 5 clearly visualizes the effect for the bent Ir sample in this manner as it is possible to distinguish three intensity minima from the thickness oscillations that are spread over an extended detector area, much larger than the direct beam.

**Figure 6.**
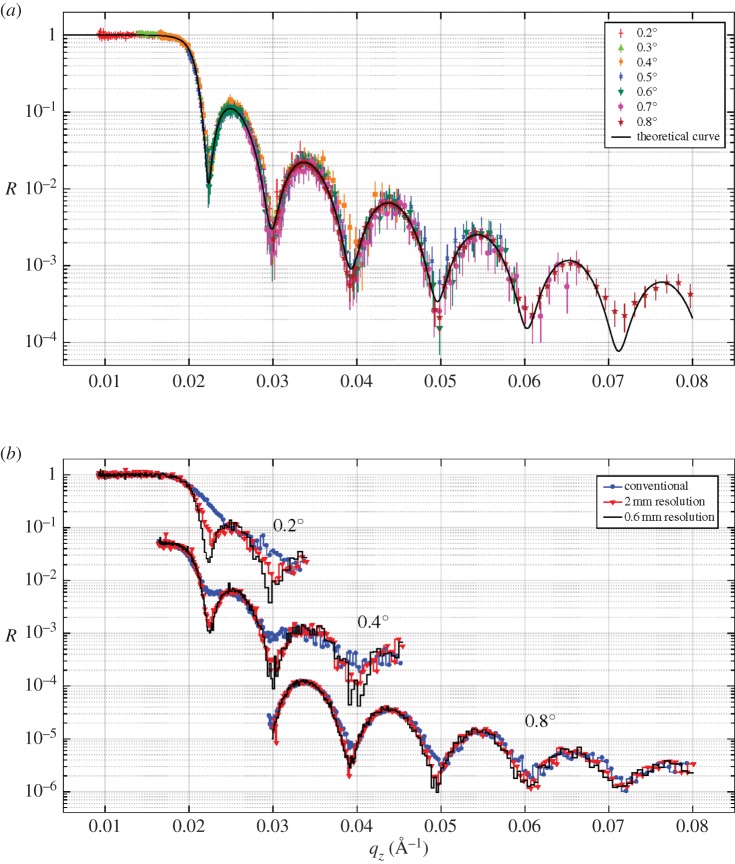
Reflectivity curves (*R*) as a function of the wavevector transfer (*q*_*z*_) from an Ir sample measured with the Multi-Blade detector at several angles and fits (*a*). Reflectivity curves for the three angles of 0.2°, 0.4° and 0.8° using the conventional analysis and the *θ*-corrected analysis with two spatial resolutions of the detector, 0.6 mm and 2 mm (*b*).

The sample is a layer of Ir of 550 Å deposited on a Si substrate. The roughness between the two interfaces is ≈ 10 Å with scattering length density *N*_*b*_ = 7.3 × 10^−6^Å^−2^ (see equation (1.2)). Figure 6*a* shows the reflectivity curves for several angles used in the measurement, in the range 0.2–0.8°, in steps of 0.1°. The theoretical reflectivity is also shown; it is calculated using the Parratt formalism [39] and is in good agreement with the experimental data.

A comparison between the conventional analysis and the *θ*-corrected reduction is shown in figure 6*b* for the three angles: 0.2°, 0.4° and 0.8°. The *θ*-correction was applied considering two pixel sizes, the actual Multi-Blade resolution and a reduced ≈ 2 mm resolution, which is the current limit of state-of-art detectors used in neutron reflectometry.

At smaller angles, the *q*-resolution depends on the detector spatial resolution to a larger extent. By applying the conventional analysis, the fringes at 0.2° and 0.4° are less visible than if the *θ*-corrected analysis is used as the better spatial resolution of the detector leads to deeper fringes.

### (b) Specular reflectometry on Si sample: dynamic range, collimated and divergent modes

The aim of the measurements presented in this section is to demonstrate the Multi-Blade detector's capabilities in a set-up as similar as possible to the ESTIA working configurations as described in §3. The instrument was operated in two configurations (collimated and divergent modes; figure 2) and measured the specular reflectivity from a Si sample.

The collimated mode is the conventional working configuration of a reflectometer, where the divergence of the beam is limited due to the slit settings and typically its contribution to the *q*-resolution is set similar to the λ contribution.

On the other hand, the divergent mode exploits the full divergence available at the instrument by only constraining those parts of the beam that would not impinge on the sample with the slits. The position of the neutron on the detector is used to encode *θ* in a similar manner to that described in the previous section, according to equation (4.2). Now the factor *f* is not needed as the sample surface is flat and the change in reflection angle corresponds to the same change in incidence angle. By allowing a wider divergence of the beam, the sampled *θ*-range is also larger; the available flux at the sample increases and thus the measuring time is reduced. This method for data reduction refers to the one that will be used with ESTIA to allow measurement from very small samples. A detailed description is reported in [14].

Although the geometry used for these measurements on CRISP is only an approximate reproduction of the focusing concept used in ESTIA [14], it is useful to test the effectiveness of the Multi-Blade detector response. Note that the focusing obtained with the slits instead of a focusing guide leads to a lower signal and a higher background as the available divergence is smaller and the sample area is strongly over-illuminated [14].

The measurement of specular reflectivity was performed in either configuration on a Si sample at three angles (0.2°, 0.3°, 0.8°). A further measurement at 1.2° was performed for the divergent mode to reach a wider dynamic range.

**Figure 7.**
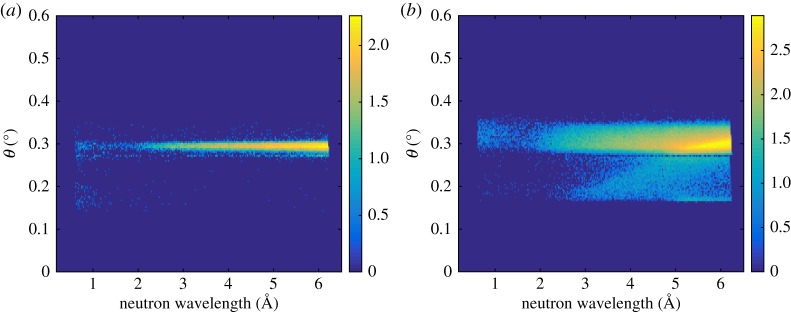
The (*θ*, λ) space for the reflectivity of the Si sample at 0.3° in the collimated (*a*) and divergent (*b*) configuration. The colour bar represents counts on a logarithmic scale.

In figure 7, the intensities of the beam in the (*θ*, λ) space in the collimated (figure 7*a*) and the divergent (figure 7*b*) modes are shown. The illuminated area of the detector is about five times larger in the divergent mode than in the collimated mode.

**Figure 8.**
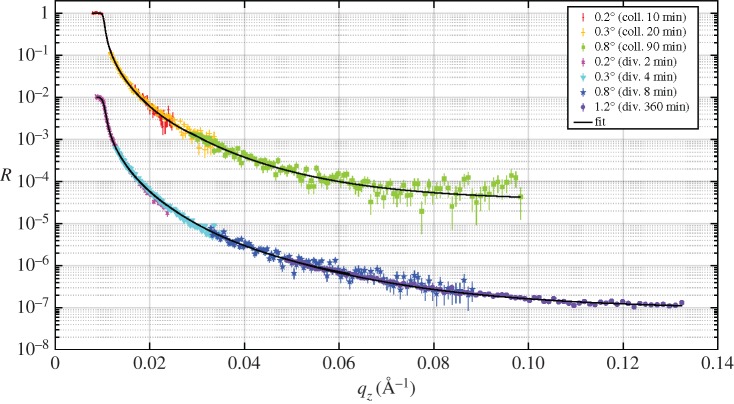
Specular reflectivity (*R*) as a function of the wavevector transfer (*q*_*z*_) of the Si sample obtained with the collimated and divergent modes. The curves obtained with the divergent mode are shifted by 0.01 in R for clarity.

Figure 8 depicts the extracted reflectivity of the sample in the two configurations. The fit used a model with Si topped with an SiO_2_ layer of 5.1 Å for both measurements and a roughness of ≈ 3 Å. Also a constant background was fitted for each angle. A relative wavelength resolution of 3% was applied; and a constant resolution of 4 × 10^−4^Å^−1^ was used for the divergent case, which is only relevant at the smallest angle. This roughly corresponds to 0.7 mm FWHM detector resolution. The total acquisition time for the three angles in the collimated mode is 120 min. The same result is obtained in 14 min by performing the measurements in the divergent mode. The acquisition time is thus improved by about one order of magnitude. Despite the high background due to the poor shielding of the Multi-Blade set-up on CRISP, a dynamic range of ≈4 orders of magnitude with the three angles was achieved. With a further measurement at 1.2°, we achieved one extra order of magnitude in the dynamic range, which is shown in figure 8. Five orders of magnitude is the dynamic range typically reached on this instrument [40]. It is expected that a deeper dynamic range in a better shielded instrument-operating environment can be measured with the Multi-Blade.

### (c) Off-specular scattering: Fe/Si super-mirror sample

**Figure 9.**
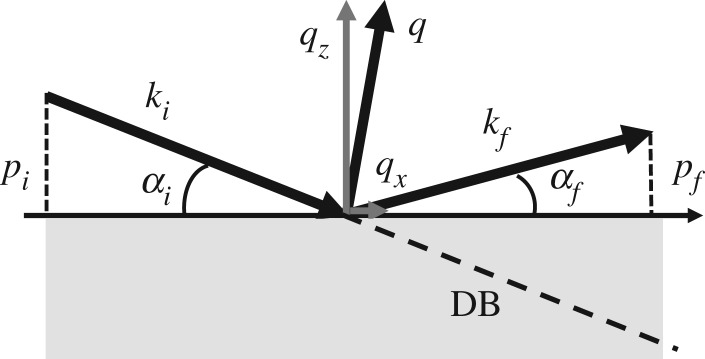
Sketch of the wave vectors' definition used in the off-specular scattering.

The specular reflectivity allows the structure of a sample to be probed across its depth; indeed the scattering vector *q* is perpendicular to the sample surface. It is possible to probe the in-plane structure of a sample by introducing a small parallel component of the scattering vector [18]; a sketch is shown in figure 9. The parameter used to reproduce the results of the off-specular scattering are the components of *q* and the projections of the initial and final wave vectors:

4.3 $$\begin{array}{rl} p_{i} & =\frac{2\pi }{\lambda }\sin\;\alpha_{i},\\ p_{f} & =\frac{2\pi }{\lambda }\sin\;\alpha_{f},\\ q_{x} & =\frac{2\pi }{\lambda }(\cos\;\alpha_{f}-\cos\;\alpha_{i})\\ \;\text{and}\;q_{z} & =\frac{2\pi }{\lambda }(\sin\;\alpha_{f}+\sin\;\alpha_{i}). \end{array}\}$$

Neutron off-specular scattering probes the in-plane structure at the micrometre length scale. The limitation of this technique is set by both the limited available neutron flux and the small scattering probability. Similarly, correlations at the nanometre length scale can be reached with a collimated beam in both directions, so-called grazing incidence small-angle scattering (GISANS), which is described in detail in [17,41–43]. On magnetic samples the off-specular technique allows the depth-resolved measurement of correlations from magnetic domains as used in [44,45].

Several specific areas can be identified in the off-specular scattering, based on the direction of the final wavevector determined by the reflected angle [46]. The horizon is defined as *α*_*f*_ = 0, when the neutron beam is parallel to the surface of the sample. The specular reflection is found at *α*_*i*_ = *α*_*f*_ and all other areas above the horizon mark the off-specular scattering region. The direct beam, DB in figure 9, meets the condition *α*_*f*_ =  − *α*_*i*_. When the incident angle is close to the critical angle *α*_*c*_, the transmitted beam is also refracted and thus this equality does not hold for small *α*_*i*_. Finally, at *α*_*i*_ = *α*_*c*_ and *α*_*f*_ = *α*_*c*_ one finds the so-called Yoneda wings, which are the result of dynamic effects mostly produced from surface roughness and magnetic domains.

The sample employed to carry out the measurements was a super-mirror Fe/Si (*m* = 3.8). It shows a strong off-specular scattering when un-magnetized because of complex magnetic domain structures. The off-specular measurements are used to test the detector performance. Good uniformity and spatial resolution as well as large dynamic range are needed to fully characterize the features of the off-specular scattering on the sample.

**Figure 10.**
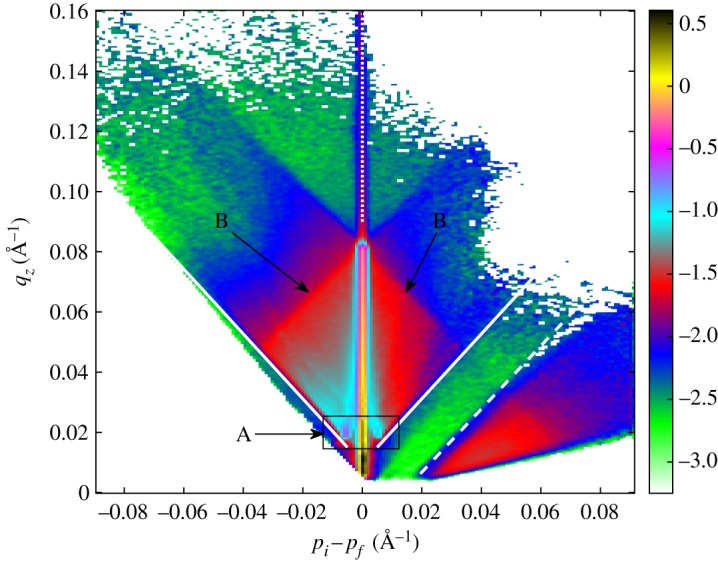
Off-specular scattering, expressed as *q*_*z*_ as a function of *p*_*i*_ − *p*_*f*_, from the Fe/Si super-mirror: the solid lines correspond to the two Yoneda wings, the dashed line denotes the beginning of a region of scattered neutrons in the transmission direction (anti-Yoneda), while the dotted line marks the specular reflectivity. The spin flip signal from the layers is highlighted with the rectangle indicated with A. The correlated domains from the sample layers correspond to the blue and red rhombus-shaped area (B). The colour scale represents counts in logarithmic units.

We performed some measurements using the collimated beam to scan the sample in angle in order to fine-tune the uniformity and reach a wider *q*-space. The sample position was tilted in steps of 0.01° in the 0.2–0.8° range. The measurements were performed during a whole night. The data are presented in the (*p*_*i*_ − *p*_*f*_, *q*_*z*_) coordinates in figure 10 and the typical features of the sample are well reproduced. In figure 10, the solid lines correspond to the two Yoneda wings, the dashed line denotes the beginning of a region of scattered neutrons in the transmission direction (anti-Yoneda), while the dotted line marks the specular reflectivity. In the specular reflectivity direction both Si and the super-mirror edge are identified (black line and the crossing point on the top of the line, respectively). The correlated domains from the sample layers correspond to the red and light blue rhombus-shaped areas. This almost featureless area corresponds to magnetic spin-flip scattering within the super-mirror that can be separated by polarization analysis into two asymmetric components, as is demonstrated in [47]. Neither beam polarization nor a magnetic field has been employed, therefore the magnetic scattering of all spin states are summed together, resulting in the rhombus area. Nevertheless, the test demonstrates the establishment of the Multi-Blade detector technology for neutron reflectometry application.

## 5. Conclusion and outlook

The neutron reflectometry technique represents a challenge in terms of instrument design and detection performance. Nowadays, several methods have been proposed to increase the incoming flux, leading to improvements for specular neutron reflectivity measurements. Along with the instruments' operation the detectors' response must be refined. The current detector technology is limited mainly with regard to spatial resolution and counting rate capability. The Multi-Blade detector has been proposed as a valid alternative to replace state-of-art detectors, because of the better performance on both spatial resolution and counting rate capability. The requirements for this technology are set for the ESS reflectometers (ESTIA [12–14] and FREIA [10,11]). Hence, apart from the ESS reflectometers, reflectometers at other facilities can take advantage of employing the Multi-Blade detector technology.

A campaign of scientific measurements has been performed on the CRISP [33] reflectometer at ISIS (Science & Technology Facilities Council in the UK [34]). The reflectivity of several reference samples has been measured by operating the instrument in various configurations to reproduce the set-up that will be used at the ESS reflectometers. Not only do the measurements provide a validation of the Multi-Blade as a mature technology for neutron reflectometry experiments, but also it has been shown that the instrument operation was improved using the Multi-Blade.

The spatial resolution of a detector is, indeed, strongly connected to the achievable *q*-resolution of the instrument. The calculated *q*_*z*_ is a combination of the neutron wavelength and the scattering angle; the latter can be corrected by taking into account the spatial resolution of the detector and thus a higher *q*-resolution is achieved. When measuring the specular reflectivity from a sample which shows interference fringes in *q*_*z*_, such as the Ir on Si, the fringes become more visible as the spatial resolution of the detector improves. The result has been compared with a conventional non-position-sensitive detector and with a state-of-the-art detector with 2 mm resolution.

It has been shown that the CRISP instrument can be operated in the REFocus mode [23] (divergent mode) by employing a position-sensitive detector. This is one of the standard modes foreseen for the ESTIA reflectometer [14] at ESS. In this configuration, the correction of the scattering angle for calculating *q*_*z*_ is mandatory and the spatial resolution and the counting rate capability of the detector are key features. This operation mode benefits from the improved spatial resolution of the Multi-Blade detector.

From the measurements of the Si sample, the *q*-range was measured to five orders of magnitude, reaching the limits of the instrument, despite the high background at the CRISP instrument and the poor shielding of the Multi-Blade detector.

An off-specular scattering measurement was also performed on a super-mirror Fe/Si multilayer sample. Neither beam polarization nor a magnetic field has been used in order to have a strong off-specular scattering from the sample. The ability of the Multi-Blade to measure not only specular, but also off-specular scattering was shown.

The results presented here show that the Multi-Blade detector technology is mature, and ready for implementation on neutron reflectometers.

## Data accessibility

The raw reflectometry data (doi:10.6084/m9.figshare.6138986) of the experiment described in this manuscript can be downloaded from https://figshare.com/s/2712231a48e19818c0c9.
